# Supplementary material for: The Vps13-like protein BLTP2 regulates phosphatidylethanolamine levels to maintain plasma membrane fluidity and breast cancer aggressiveness
Source: Nat Cell Biol. 2025 Jun 27;27(7):1125–35. doi: 10.1038/s41556-025-01672-3 (PMC12270902; doi:10.1038/s41556-025-01672-3)
Supplement: Supplementary file 1 — Reporting Summary [file 41556_2025_1672_MOESM1_ESM.pdf]

nature portfolio

Corresponding author(s): William A. Prinz, Alexandre Toulmay

Last updated by author(s): Feb 21, 2025

Reporting Summary

Nature Portfolio wishes to improve the reproducibility of the work that we publish. This form provides structure for consistency and transparency in reporting. For further information on Nature Portfolio policies, see our Editorial Policies and the Editorial Policy Checklist.

Statistics

For all statistical analyses, confirm that the following items are present in the figure legend, table legend, main text, or Methods section.

n/a	Confirmed	
☐	☒	The exact sample size (*n*) for each experimental group/condition, given as a discrete number and unit of measurement
☐	☒	A statement on whether measurements were taken from distinct samples or whether the same sample was measured repeatedly
☐	☒	The statistical test(s) used AND whether they are one- or two-sided<br>*Only common tests should be described solely by name; describe more complex techniques in the Methods section.*
☒	☐	A description of all covariates tested
☐	☒	A description of any assumptions or corrections, such as tests of normality and adjustment for multiple comparisons
☐	☒	A full description of the statistical parameters including central tendency (e.g. means) or other basic estimates (e.g. regression coefficient) AND variation (e.g. standard deviation) or associated estimates of uncertainty (e.g. confidence intervals)
☐	☒	For null hypothesis testing, the test statistic (e.g. F, t, r) with confidence intervals, effect sizes, degrees of freedom and P value noted<br>*Give P values as exact values whenever suitable.*
☒	☐	For Bayesian analysis, information on the choice of priors and Markov chain Monte Carlo settings
☒	☐	For hierarchical and complex designs, identification of the appropriate level for tests and full reporting of outcomes
☒	☐	Estimates of effect sizes (e.g. Cohen's *d*, Pearson's *r*), indicating how they were calculated

Our web collection on statistics for biologists contains articles on many of the points above.

Software and code

Policy information about availability of computer code

Data collection	Commercially available software was used to collect data. UVP Gels solo Visionworks 9.1.20063.7760, Raytest Rita TLC control RS232, Raytest Gina Star TLC v.5.01, Agilent OpenLAB CDS ChemStation v2.19.20, Agilent MassHunter WorkStation v10.1, GE DeltaVision Cytiva Softworx, NIS elements AR5.42.0, Licor Odyssey Image Studio5.2.2, Licor acquisition v1.2.0.72.
Data analysis	Either open sourced or commercially available softwares were used to collect data; Microsoft Excel for Mac Version 16.94, Graphpad Prism 10, FiJi (ImageJ2 v2.9.0/1.53t), lmaris (CF package, v10.2.0, Oxford Instruments), GE DeltaVision Cytiva Softworx, Nikon (NIS) elements AR Analysis5.42.06 64-bit, BD FACSDiva 8.0.2

For manuscripts utilizing custom algorithms or software that are central to the research but not yet described in published literature, software must be made available to editors and reviewers. We strongly encourage code deposition in a community repository (e.g. GitHub). See the Nature Portfolio guidelines for submitting code & software for further information.

Data

Policy information about availability of data

All manuscripts must include a data availability statement. This statement should provide the following information, where applicable:

- Accession codes, unique identifiers, or web links for publicly available datasets
- A description of any restrictions on data availability
- For clinical datasets or third party data, please ensure that the statement adheres to our policy

Data used in this study are available in the main figures, extended data figures, source data and supplementary tables of this article. Publicly available CRISPR Chronos data set was taken from DepMap.

Research involving human participants, their data, or biological material

Policy information about studies with human participants or human data. See also policy information about sex, gender (identity/presentation), and sexual orientation and race, ethnicity and racism.

Reporting on sex and gender	n/a
Reporting on race, ethnicity, or other socially relevant groupings	n/a
Population characteristics	n/a
Recruitment	n/a
Ethics oversight	n/a

Note that full information on the approval of the study protocol must also be provided in the manuscript.

Field-specific reporting

Please select the one below that is the best fit for your research. If you are not sure, read the appropriate sections before making your selection.

☒ Life sciences ☐ Behavioural & social sciences ☐ Ecological, evolutionary & environmental sciences

For a reference copy of the document with all sections, see nature.com/documents/nr-reporting-summary-flat.pdf

Life sciences study design

All studies must disclose on these points even when the disclosure is negative.

Sample size	No statistical method was used to predetermine sample size.
Data exclusions	No data were excluded.
Replication	Graphical data and representative immunoblots are from at least three independent replicates. Micrographs are from two to three independent repeats. All attempts at replication were successful.
Randomization	No randomization was used as this study does not involve human participants.
Blinding	Investigators were not blinded to group allocation. Blinding was not required because the study does not have analysis of data from human participants.

Reporting for specific materials, systems and methods

We require information from authors about some types of materials, experimental systems and methods used in many studies. Here, indicate whether each material, system or method listed is relevant to your study. If you are not sure if a list item applies to your research, read the appropriate section before selecting a response.

Materials & experimental systems

n/a	Involved in the study
☐	☒ Antibodies
☐	☒ Eukaryotic cell lines
☒	☐ Palaeontology and archaeology
☐	☒ Animals and other organisms
☒	☐ Clinical data
☒	☐ Dual use research of concern
☒	☐ Plants

Methods

n/a	Involved in the study
☒	☐ ChIP-seq
☐	☒ Flow cytometry
☒	☐ MRI-based neuroimaging

Antibodies

Antibodies used

Antibodies used in this study, their description, and dilutions used are listed in supplementary table 2 in the article.

Validation

All commercially available antibodies are prevalidated by the manufacturing company. Anti Pma1 antibody was validated in PMID: 29254995 (for Pma1) , and anti Ypt7 antibody was validated by the Dr. William Wickner lab at Dartmouth in PMID: 12177043.

1. CST anti-HA
"Specificity / Sensitivity
HA-Tag (C29F4) Rabbit mAb detects exogenously expressed proteins containing the HA epitope tag. The antibody may cross-react with a protein of unknown origin ~100kDa.

Species Reactivity:

All Species Expected"

2. Thermofisher sientific anti-Porin (459500)

"The antibody was verified by Cell treatment to ensure that the antibody binds to the antigen stated."

3. Thermofisher scientific anti-Dpm1 (A6429)
The manufacturer confirms this antibody is a mouse monoclonal IgG1 (clone number (5C5A7) which reacts to yeast Dpm1p.

4. Abcam anti-Vph1 (10D7A7B2)
The manufacturer confirms that the mouse monoclonal IgG2a antibody (clone number (10D7A7B2) antibody reacts with yeast Vph1p.

5. CST Anti-Caveolin-1 (D46G3)

"Specificity / Sensitivity
Caveolin-1 (D46G3) XP® Rabbit mAb detects endogenous levels of total caveolin-1 protein.

Species Reactivity:

Human, Mouse, Rat, Hamster, Monkey, Bovine, Dog"

6. CST anti-GAPDH antibody (14C10)

"Specificity / Sensitivity
GAPDH (14C10) Rabbit mAb detects endogenous levels of total GAPDH protein.

Species Reactivity:

Human, Mouse, Rat, Monkey, Bovine, Pig"

7. CST Anti-VDAC antibody (D73D12)

"Specificity / Sensitivity
VDAC (D73D12) Rabbit mAb detects endogenous levels of total VDAC protein.

Species Reactivity:

Human, Mouse, Rat, Monkey"

8. CST Anti-PDI antibody (C81H6)

"Specificity / Sensitivity
PDI (C81H6) Rabbit mAb detects endogenous levels of total PDI protein.

Species Reactivity:

Human, Mouse, Rat, Monkey"

9. IRDye 800CW anti-Rabbit IgG (LI-COR 926-32211)

"Highly cross-adsorbed goat (polyclonal) anti-rabbit IgG (H+L) antibody conjugated to IRDye 800CW.

Immunogen
Rabbit IgG

Purity and Specificity
Isolation of specific antibodies was accomplished by affinity chromatography using pooled rabbit IgG covalently linked to agarose. Based on ELISA and flow cytometry, this antibody reacts with the heavy and light chains of rabbit IgG, and with the light chains of rabbit IgM and IgA. This antibody was tested by dot blot and and/or solid-phase adsorbed for minimal cross-reactivity with human, mouse, rat, sheep, and chicken serum proteins, but may cross-react with immunoglobulins from other species. The conjugate has been specifically tested and qualified for Western blot and In-Cell Western™ Assay applications."

10. IRDye 680RD anti-mouse IgG (LI-COR 926-68070)

"Highly cross-adsorbed goat (polyclonal) anti-mouse IgG (H+L) antibody conjugated to IRDye 680RD.

Immunogen
Mouse IgG paraproteins

Purity and Specificity
Isolation of specific antibodies was accomplished by affinity chromatography using pooled mouse IgG covalently linked to agarose. Based on ELISA and flow cytometry, this antibody reacts with the heavy and light chains of mouse IgG1, IgG2a, IgG2b, and IgG3, and with the light chains of mouse IgM and IgA. This antibody was tested by dot blot and and/or solid-phase adsorbed for minimal cross-reactivity with human, rabbit, goat, rat, and horse serum proteins, but may cross-react with immunoglobulins from other species. The conjugate has been specifically tested and qualified for Western blot and In-Cell Western™ Assay applications."

Eukaryotic cell lines

Policy information about cell lines and Sex and Gender in Research

Cell line source(s)	S. cerevisiae cell lines were either collected from the non-essential gene deletion library or constructed in the Prinz lab; Mammalian Cell lines (HeLa, MDA-MB-231, and MCF10A) were purchased from ATCC. HeLa cells with TLCD1 deletion and isogenic untargeted guide RNA transfected containing control HeLa cell lines were a gift from the Kasparas Petkevicius lab in Cambridge University, UK.
Authentication	S. cerevisiae cell lines were authenticated by PCR using primers against S. cerevisiae genome, selecting against selection markers, immunoblotting, and crossing . Mammalian cell lines were authenticated by ATCC. Cell lines gifted by the Petkevicius lab were authenticated by their lab by PCR using human genome specific primers.
Mycoplasma contamination	Not contaminated. Assessed by MycoAlert Mycoplasma Detection Kit (Lonza Cat# LT07-118).
Commonly misidentified lines (See ICLAC register)	Commonly misidentified cell lines were not used in this study

Animals and other research organisms

Policy information about studies involving animals; ARRIVE guidelines recommended for reporting animal research, and Sex and Gender in Research

Laboratory animals	Only animals used in this study is the zebrafish Danio rerio. Details about Zebrafish husbandry is mentioned in the materials and methods section.

Wild animals | n/a

Reporting on sex | Sex of zebrafish was not considered in this study.

Field-collected samples | n/a

Ethics oversight | n/a

Note that full information on the approval of the study protocol must also be provided in the manuscript.

Plants

Seed stocks | n/a

Novel plant genotypes | n/a

Authentication | n/a

Flow Cytometry

Plots

Confirm that:

- [x] The axis labels state the marker and fluorochrome used (e.g. CD4-FITC).
- [x] The axis scales are clearly visible. Include numbers along axes only for bottom left plot of group (a 'group' is an analysis of identical markers).
- [x] All plots are contour plots with outliers or pseudocolor plots.
- [x] A numerical value for number of cells or percentage (with statistics) is provided.

Methodology

Sample preparation | Mentioned in the materials and methods section. Samples were prepared following the manufacturer's protocol of the Annexin-V apoptosis assay kit (Thermofisher Scientific Cat. No. V13241).

Instrument | BD FACS Flow Cytometer

Software | BD FACSDiva 8.0.2

Cell population abundance | Cells were from homogenous cultures, unlike mixed cell types from a tissue. Therefore, this field is not applicable.

Gating strategy | A schematic figure explaining the gating strategy for the experiment is provided as Extended data Fig. 9. A representative flow cytometry figure is in Extended data fig. 10.

- [x] Tick this box to confirm that a figure exemplifying the gating strategy is provided in the Supplementary Information.
