# Supplementary material for: The Vps13-like protein BLTP2 regulates phosphatidylethanolamine levels to maintain plasma membrane fluidity and breast cancer aggressiveness
Source: Nat Cell Biol. 2025 Jun 27;27(7):1125–35. doi: 10.1038/s41556-025-01672-3 (PMC12270902; doi:10.1038/s41556-025-01672-3)
Supplement: Supplementary file 4 — Western blots. [file 41556_2025_1672_MOESM4_ESM.pdf]

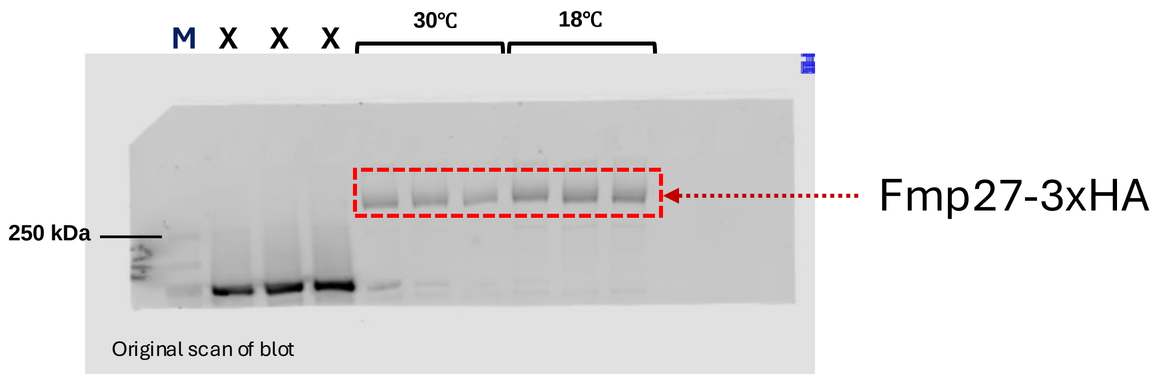

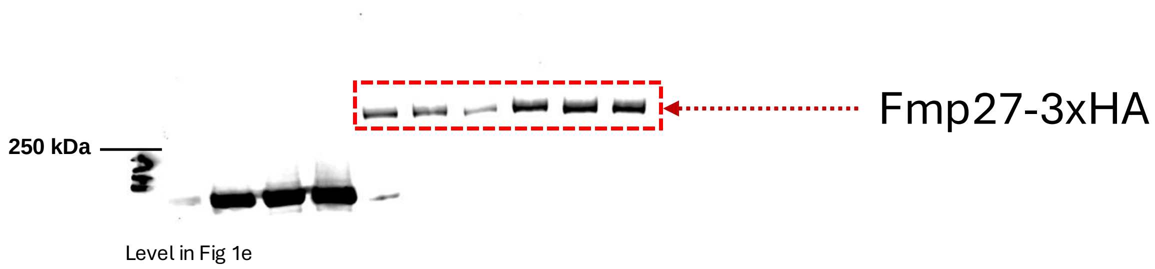

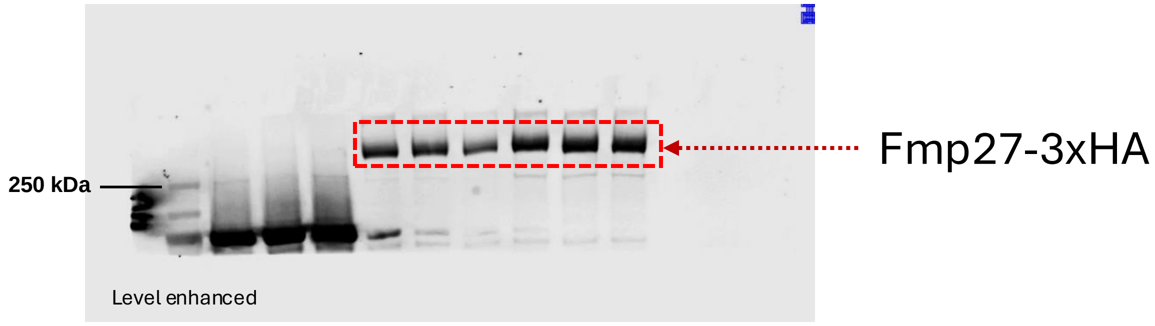

Dashed lines indicate region shown in main figure.
M = molecular weight markers.
X = lanes with samples not included in main figure.

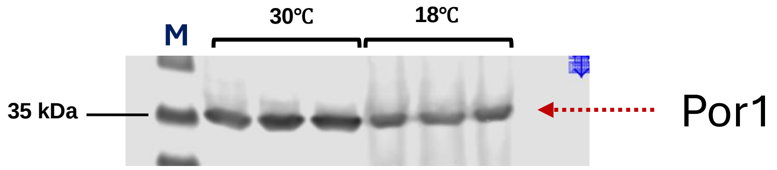

Source Data for Fig. 1
