# Supplementary material for: The Vps13-like protein BLTP2 regulates phosphatidylethanolamine levels to maintain plasma membrane fluidity and breast cancer aggressiveness
Source: Nat Cell Biol. 2025 Jun 27;27(7):1125–35. doi: 10.1038/s41556-025-01672-3 (PMC12270902; doi:10.1038/s41556-025-01672-3)
Supplement: Supplementary file 5 — Western blots. [file 41556_2025_1672_MOESM5_ESM.pdf]

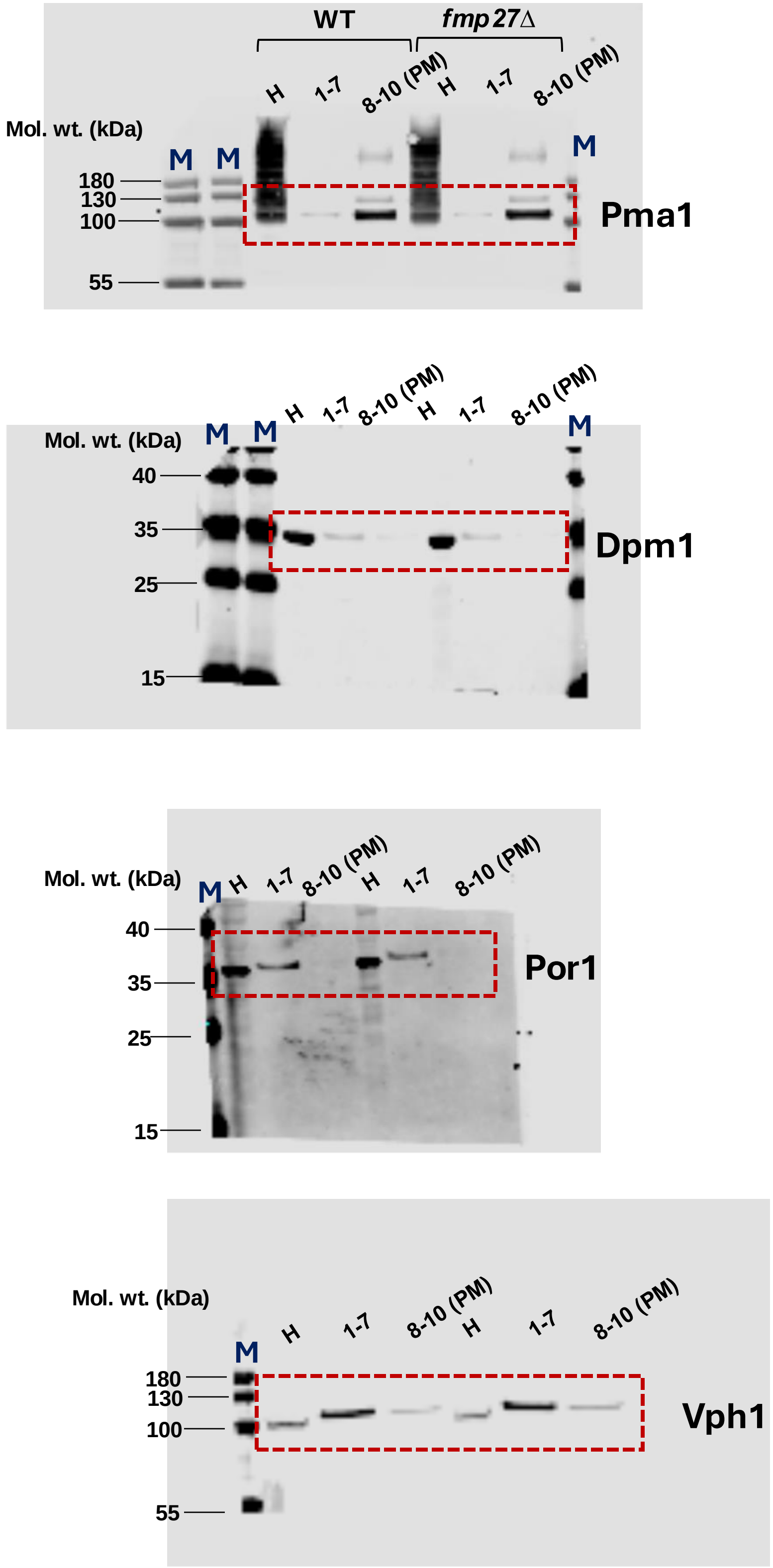

Dashed lines indicate portions shown in main figures.

Source Data for Fig. 2b

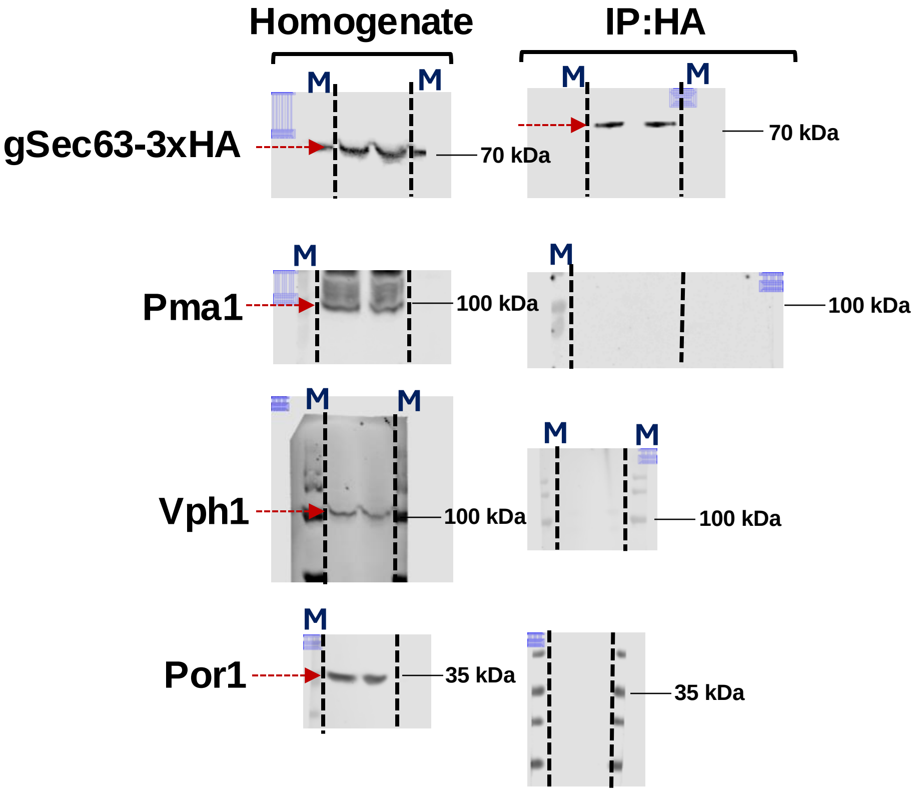

Dashed lines indicate separation between lanes with protein samples and molecular weight markers

Source Data for Fig. 2e
