# Supplementary figures and images for: The Vps13-like protein BLTP2 regulates phosphatidylethanolamine levels to maintain plasma membrane fluidity and breast cancer aggressiveness
Source: Nat Cell Biol. 2025 Jun 27;27(7):1125–35. doi: 10.1038/s41556-025-01672-3 (PMC12270902; doi:10.1038/s41556-025-01672-3)

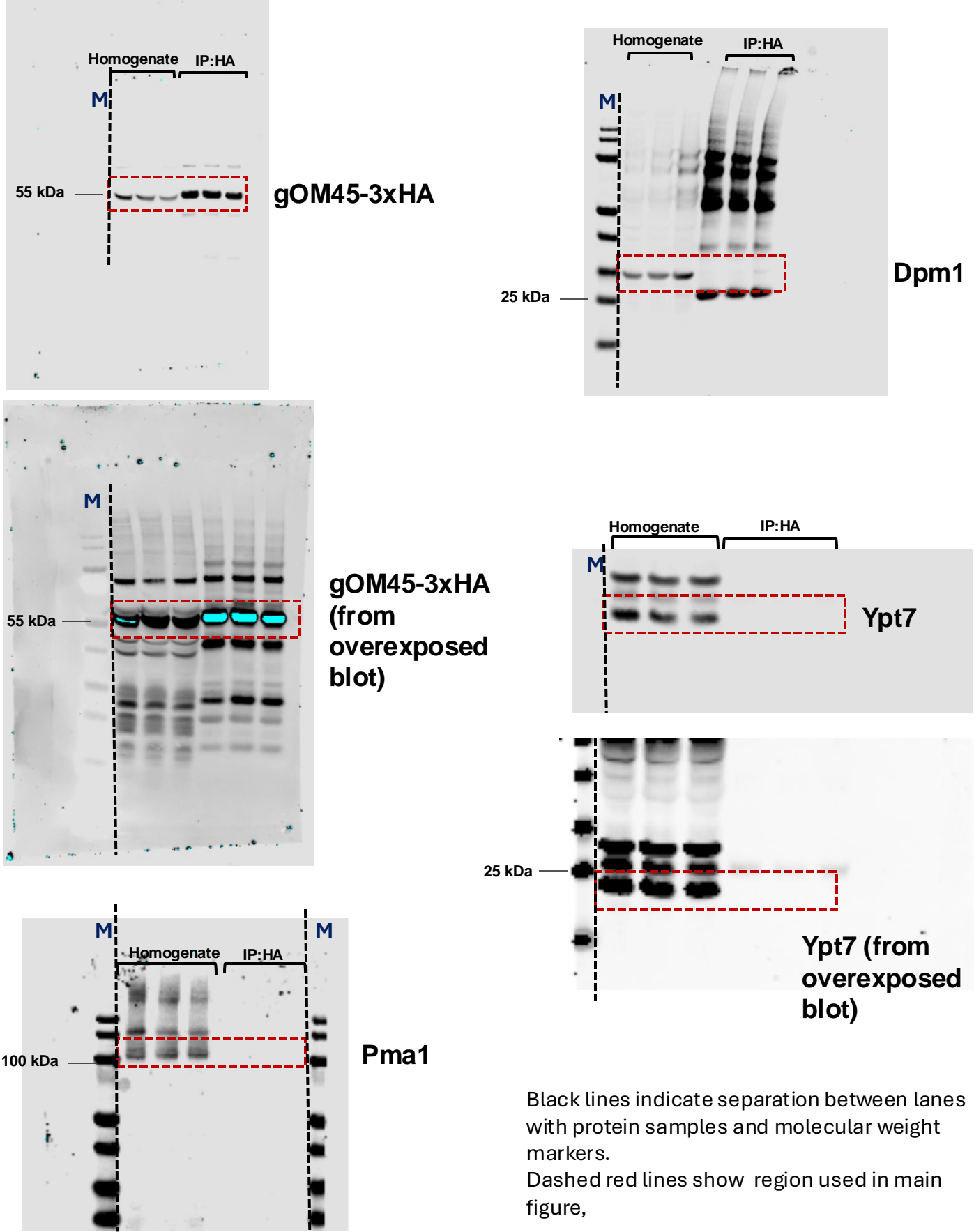

Source Data for Fig. 3h

Supplement: Supplementary file 6 — Western blots. [file 41556_2025_1672_MOESM6_ESM.pdf]

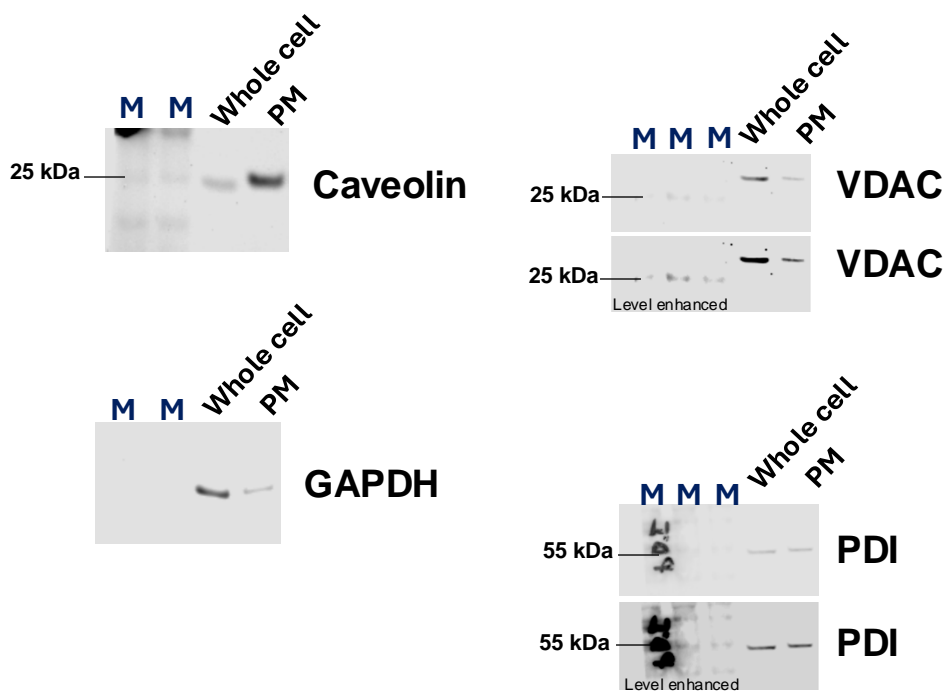

Source Data for Fig. 4d

Supplement: Supplementary file 7 — Western blots. [file 41556_2025_1672_MOESM7_ESM.pdf]
